# Load Prediction in Double-Channel Residual Self-Attention Temporal Convolutional Network with Weight Adaptive Updating in Cloud Computing

**DOI:** 10.3390/s24103181

**Published:** 2024-05-17

**Authors:** Jiang Lin, Yepeng Guan

**Affiliations:** 1 School of Communication and Information Engineering, Shanghai University, Shanghai 200444, China; linjiang10001@shu.edu.cn; 2 Key Laboratory of Advanced Display and System Application, Ministry of Education, Shanghai 200072, China; 3 Key Laboratory of Silicate Cultural Relics Conservation, Shanghai University, Ministry of Education, Shanghai 200444, China

**Keywords:** cloud computing load prediction, residual temporal attention mechanism, double-channel temporal convolutional network, adaptive weight update

## Abstract

When resource demand increases and decreases rapidly, container clusters in the cloud environment need to respond to the number of containers in a timely manner to ensure service quality. Resource load prediction is a prominent challenge issue with the widespread adoption of cloud computing. A novel cloud computing load prediction method has been proposed, the Double-channel residual Self-attention Temporal convolutional Network with Weight adaptive updating (DSTNW), in order to make the response of the container cluster more rapid and accurate. A Double-channel Temporal Convolution Network model (DTN) has been developed to capture long-term sequence dependencies and enhance feature extraction capabilities when the model handles long load sequences. Double-channel dilated causal convolution has been adopted to replace the single-channel dilated causal convolution in the DTN. A residual temporal self-attention mechanism (SM) has been proposed to improve the performance of the network and focus on features with significant contributions from the DTN. DTN and SM jointly constitute a dual-channel residual self-attention temporal convolutional network (DSTN). In addition, by evaluating the accuracy aspects of single and stacked DSTNs, an adaptive weight strategy has been proposed to assign corresponding weights for the single and stacked DSTNs, respectively. The experimental results highlight that the developed method has outstanding prediction performance for cloud computing in comparison with some state-of-the-art methods. The proposed method achieved an average improvement of 24.16% and 30.48% on the Container dataset and Google dataset, respectively.

## 1. Introduction

With the widespread adoption of cloud computing technology, many enterprises choose to migrate their business to the cloud for greater flexibility and scalability [1]. In the context of cloud computing, it is very important to plan and utilize resources rationally. Both server capacity and resources can be better allocated to meet the diverse needs of customers. Load prediction plays a vital role as a technique that enables businesses to predict future resource needs [2]. Applying accurate load prediction results to resource allocation plays a key role in enterprise resource utilization. Accurate load prediction helps to optimize the performance of cloud computing and server systems.

However, the dynamics and complexity of cloud computing environments pose several challenges to load forecasting techniques. To address these challenges, it is crucial to develop efficient and accurate time series prediction algorithms to achieve high availability and performance in cloud computing environments. The resource situation in the cloud platform can be treated as a time series, and the developed models and algorithms can be used to predict resource usage. Therefore, developing a prediction algorithm to improve the accuracy of prediction is an urgent problem that needs to be solved.

Some single-variable time series models including Autoregressive Integrated Moving Average (ARIMA) [3], linear regression [4], and Exponentiated Linear Regression (ELR) [5] are widely used for predicting stationary time series. A predictive model [6] based on ARIMA [3] was proposed for energy consumption prediction. The model was improved by proposing an ARMA [3] for time series prediction [7]. However, these models have poor performance for non-periodic time series. Additionally, they are prone to overfitting and other issues for high-dimensional and long time series.

Another approach is to use machine learning models, such as linear Support Vector Regression (SVR) [8], random forests [9], ridge regression [10], LASSO [11], SOM [12], resource scaling techniques [13], or statistical learning [14]. However, machine learning models, as mentioned above, may not be able to incorporate complex nonlinear dependencies between multiple variables in large datasets.

Deep learning network architectures have been developed to overcome these limits including Convolutional Neural Networks (CNNs) [15] and Recurrent Neural Networks (RNNs) [16,17], Long Short-Term Memory (LSTM) [18], Convolutional LSTM (ConvLSTM) [19], and Multivariate Attention LSTM-Fully Convolutional Networks (MALSTM-FCN) [20]. However, these models are time-consuming and not robust when dealing with non-periodic data predictions. LSTM [18], as a variant of RNN [16], has demonstrated excellent performance in predicting CPU usage. Nevertheless, RNN [16] suffers from the issues of vanishing and exploding gradients in time series prediction.

Other novel methods for time series forecasting are the mixed multi-scale method including empirical mode decomposition (EMD) [21], ensemble empirical mode decomposition (EEMD) [22], multi-level wavelet decomposition network (mWDN) [23], variational mode decomposition (VMD) [24], and wavelet decomposition [25]. These methods [21,22,23,24,25] decompose the data into different frequency components to facilitate prediction. However, they are not generalizable for predicting non-periodic and complex time series.

Temporal Convolutional Network (TCN) [26] has been proposed as a universal architecture for handling time series tasks. TCN stacking was used in [27] to increase the feature extraction of the sequence. However, it merely overlapped the network structures without making any structural modifications. LSTM-TCN network was utilized as a predictive model in [28]. However, it has limited ability to capture long-term dependency information. The LMD-ETS-TCN model, consisting of TCN combined with other time series models, has also demonstrated promising performance [29]. However, the input data are required to possess a certain level of stationarity and periodicity characteristics. These applications have not been investigated for handling complex data in cloud platforms [30].

When predicting time series on cloud computing resources, some model methods mentioned in the references have some specific problems and flaws. Changes in cloud computing resources are dynamic, complex and non-periodic. These methods fail to accurately predict long-term sequences, and the problem about long-term dependence of sequences in complex data has not been solved.

A novel cloud computing load prediction method has been proposed, the Double-channel residual Self-attention Temporal convolutional Network with Weight adaptive updating (DSTNW). A Double-channel Temporal convolutional Network (DTN) has been adopted to improve prediction accuracy and better capture long-term dependencies in the sequence. A residual temporal Self-attention Mechanism (SM) has been adopted to add the contribution of historical data to jumps in the process [31,32,33]. DTN and SM jointly constitute a dual-channel residual self-attention temporal convolutional network (DSTN). In addition, by evaluating the accuracy aspects of single and stacked DSTNs, an adaptive weight strategy has been developed to assign corresponding weights for single and stacked DSTNs, respectively.

Some main contributions of this paper are summarized as follows:

First, DTN was proposed to capture long-term dependencies in series. Double-channel dilated causal convolution was adopted to replace the single-channel dilated causal convolution. The developed double-channel dilated causal convolution can be applied to enhance feature extraction capability and capture dependencies within the complex sequence.

Secondly, SM was developed to improve the performance of the network and focus on features that have made significant contributions. The SM module can selectively extract the dependencies and information from DTN.

More importantly, an adaptive weight update strategy was developed to assign the corresponding weights for single and stacked DSTNs. The weight is adaptively updated according to the errors in the DSTNs.

The rest of this paper is organized as follows. The DSTNW network is presented in Section 2. Experimental results are described and discussed in Section 3 and are followed by some conclusions in Section 4.

## 2. DSTNW Network

**Figure 1:**
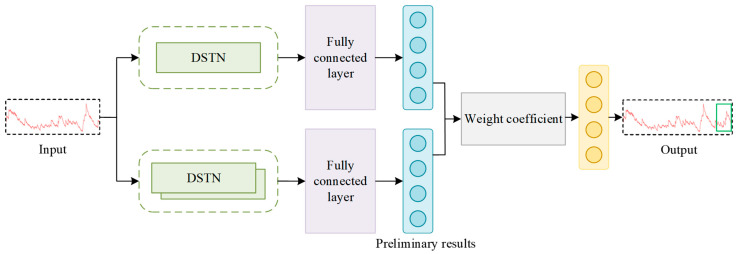
DSTNW network framework.

In this section, the details of the DSTNW network are given as shown in Figure 1. Firstly, the Double-channel Self-attention Temporal convolutional Network (DSTN) is described in Section 2.1. The DTN unit is introduced in Section 2.1.1. The SM unit is described in Section 2.1.2. The adaptive weight update strategy is discussed in Section 2.2.

### 2.1. DSTN

**Figure 2:**
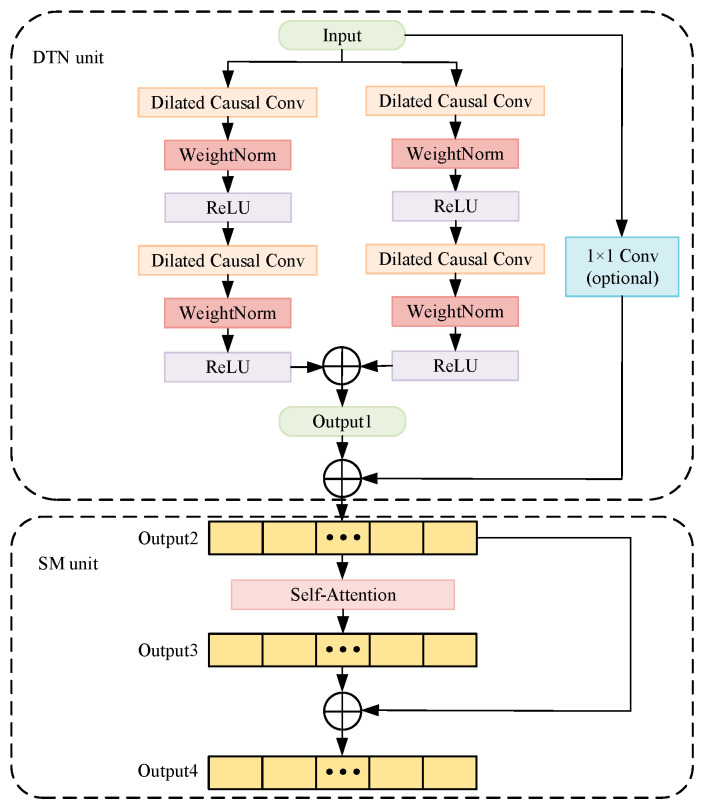
DSTN network structure.

The DSTN consists of the DTN and SM modules as shown in Figure 2.

#### 2.1.1. DTN Unit

The DTN unit is shown in the top half of Figure 2. It replaces the TCN [26] with a double-channel dilated causal convolution unit. In the DTN model, the single-channel dilated causal convolution in the TCN [26] is transformed into the double-channel dilated causal convolution. The outputs in the DTN unit are the sum of the outputs of two paths. One of the paths is the sum that the input passes through the double sides of two layers of the same dilated causal conv (DCC) and outputs. The input enters the DCC after the weights of the first layer are initialized. The output is subjected to nonlinear transformation through the Relu activation function after weight normalization. The nonlinear output is subjected to dropout regularization to reduce the over-fitting of the model. Another path is the input that goes directly to the output through a one-dimensional convolutional layer. The two paths constitute the residual block (RC), which is derived from the residual neural network. It is helpful for the construction of the deep neural network.

**Figure 3:**
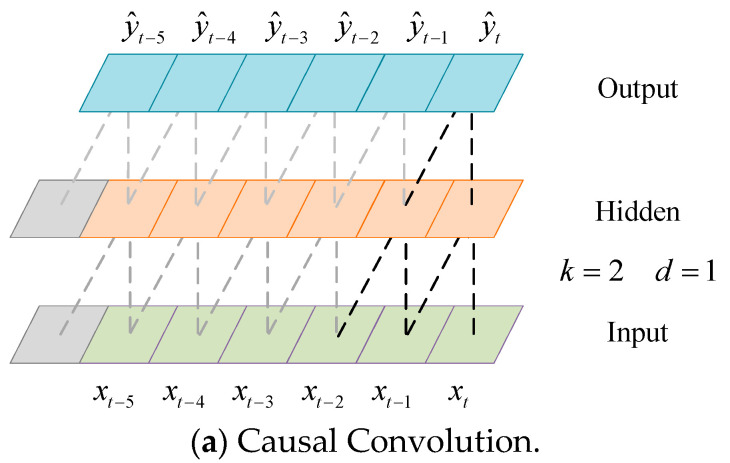

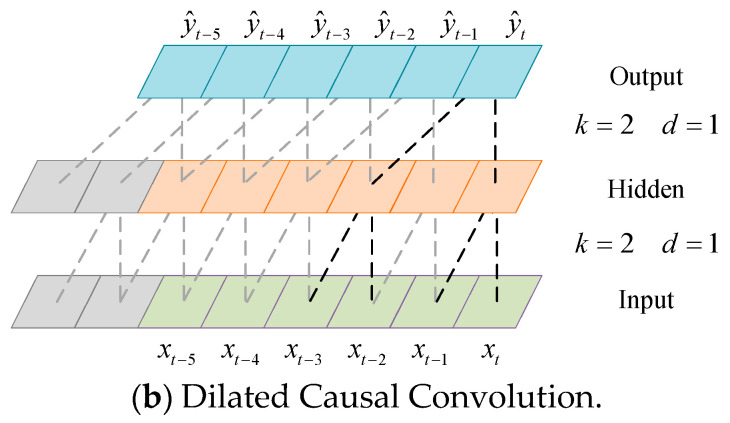
Causal Convolution and Dilated Causal Convolution.

The DCC increases the value of the expansion coefficient *d* so that it expands the receptive field of the network to accept longer historical data according to causal convolution. It is a 3-layer causal convolutional network schematic diagram as shown in Figure 3. The value of the convolution kernel *k* in this network is 2, the value of the expansion coefficient *d* is 1, and the receptive field is 3.

The convolution operation is represented by a dashed line in Figure 3. The green represents the input, blue represents the output, and orange represents the hidden layer. The predicted load sequence *ŷ_t_* is calculated from the input sequence [*x_t_*_−2_, *x_t_*_−1_, *x_t_*], and has nothing to do with the input sequence [*x_t_*_+1_, *x_t_*_+2_, …]. The application of causal convolution in the TCN [26] would not cause information leakage. Since the receptive field is small for the causal convolution in the TCN [26], the DCC is developed by increasing the expansion coefficient to expand the network receptive field as shown in Figure 3a. It can be seen from Figure 3 that the receptive field of the DCC under the same number of layers is expanded to 4.

The TCN [26] can be used to receive longer historical sequence data after applying the proposed DCC. The dilated convolution operation is shown as:

(1) $$F\left(t\right)=\sum_{v=0}^{u-1}f\left(v\right)X_{t-dv}$$

where *F*(*t*) represents the dilated convolution operation, *X_t_*_−*dv*_ is the sequence data, *f*(*v*) is the filter function, *u* is the length of the input sequence data, *v* is the value of the *v*-th element in the input sequence data, and *d* is the expansion coefficient.

Since the receptive field in this model is effectively expanded, it can acquire substantial differences and enhance the expressive power. Some long-term dependencies in the sequences can be better captured by splitting the dilated causal convolution into two parallel sides of the dilated causal convolutions.

#### 2.1.2. SM Unit

Self-attention mechanism [31,32,33] is an important improvement on traditional attention mechanisms and plays a key role in neural networks. It aims to capture the internal correlations of data and can help the model focus more on the informative information that makes a significant contribution to the output. In a time series, a self-attention mechanism is adopted to capture the features of the temporal dimension. The sequence is given different contributions on the temporal dimension by assigning different weights to each temporal element in the time series.

**Figure 4:**
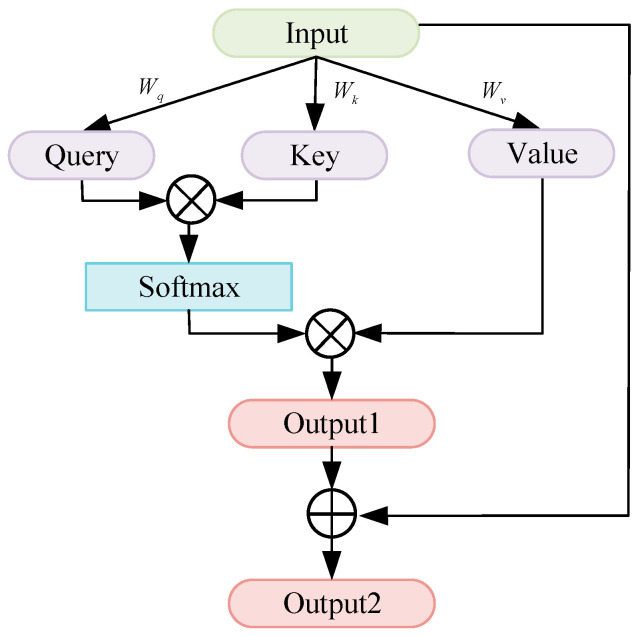
Residual temporal self-attention mechanism.

A self-attention layer is the core component of a self-attention mechanism as shown in Figure 4. The ⊕ symbol represents the addition of two values and the ⊗ symbol represents the multiplication of two values. It comprises three elements including queries, keys, and values. These three vectors are obtained by multiplying the input data with the corresponding weight matrices *W_q_*, *W_k_*, and *W_v_*, respectively:

(2) $$Q=inputs\times W_{q}$$

(3) $$K=inputs\times W_{k}$$

(4) $$V=inputs\times W_{v}$$

where × is a multiplying operator.

The result of multiplying *Q* and *K* by a ratio factor $\sqrt{d_{k}}$ is divided by a softmax function and then multiplied by *V* to obtain the output of the self-attention mechanism:

(5) $$output1=softmax\left(\frac{QK^{T}}{\sqrt{d_{k}}}\right)V$$

To address the problem of gradient disappearance and explosion in deep neural networks, a residual connection is used at the end of the temporal self-attention mechanism to prevent loss or distortion of information during the hierarchical transmission of information within the network:

(6) output2=output1+input

To better capture the relationship between the features and load sequences and obtain more important temporal information in long sequences, a residual temporal self-attention mechanism module is proposed. This module aims to capture the contributions of different elements in the sequence. The network becomes easier to optimize for enhancing the depth and accuracy of the model by connecting the residual mechanism with the self-attention mechanism. Moreover, the cross-layer connections in the residual networks can improve the performance by increasing the network depth without encountering the issues of vanishing or exploding gradients.

### 2.2. Adaptive Weight Update Strategy

Since there are some different predictive performances for single and stacked DSTNs, an adaptive weight strategy is proposed to assign the corresponding weights for the DSTNs. Some errors for single and stacked DSTNs are evaluated. Some corresponding weights are assigned adaptively to the DSTNs for each time step in the series.

**Figure 5:**
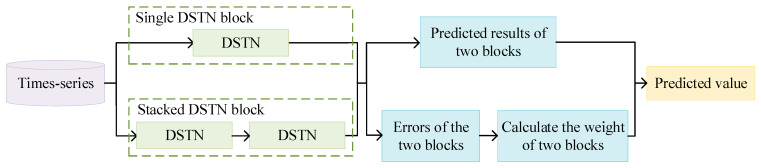
Adaptive weight update strategy framework.

Assuming that the given time step is *S*, calculate the errors for a single DSTN (block 1) and a stacked DSTN (block 2) from *t* − *s* to *t* as shown in Figure 5. The results in *error*1 and *error*2 are computed as follows:

(7) $$error1_{t}=\sum_{i=t-s+1}^{t}{\left(Pre\_block1_{t}-y_{t}\right)}^{2}$$

(8) $$error2_{t}=\sum_{i=t-s+1}^{t}{\left(Pre\_block2_{t}-y_{t}\right)}^{2}$$

where *error*1*_t_* and *error*2*_t_* are the sum of the squared prediction errors in the single DSTN (block 1) and the stacked DSTN (block 2) at *t* time. *y_i_* is the real value at *t* time. *Pre_block*1, and *Pre_block*2 are the predicted values of block 1 and block 2, respectively.

The obtained *error*1 and *error*2 are then used to calculate the corresponding weights including *weight*1 and *weight*2 for block 1 and block 2, respectively:

(9) $$weight1_{t}=\frac{error2_{t}}{error1_{t}+error2_{t}}$$

(10) $$weight2_{t}=\frac{error1_{t}}{error1_{t}+error2_{t}}$$

The weights are then applied to the corresponding time steps of block 1 and block 2 from *t* to *t* + *s*. The result for the total prediction value pre_value is as follows:

(11) $$Pre\_value1_{t+1}=weight1_{t}\times Pre\_block1_{t}$$

(12) $$Pre\_value2_{t+1}=weight2_{t}\times Pre\_block2_{t}$$

(13) $$Pre\_value_{t+1}=Pre\_value1_{t+1}+Pre\_value2_{t+1}$$

The input data are trained by two network modules in process. Two sets of predicted values from the corresponding modules obtained. Two sets of predicted values are calculated to obtain two sets of error values. The corresponding weights are calculated from the two sets of error values.

The input is passed through the single and stacked DSTNs, respectively. Some weight coefficients are obtained by the adaptive weight update strategy. The predicted results are provided in the output.

## 3. Experimental Results and Discussion

### 3.1. Datasets and Implements

All experiments were conducted with a NVIDIA GeForce GTX 1060, Intel i7-7700 CPU, and 16 GB memory to test the developed method’s performance. Some datasets were selected to perform a fair comparison with some state-of-the-art methods. Container workload traces [34] were collected from a real online Kubernetes system. The data in [34] contains 59 performance indicators collected within 30 days from an online system including CPU, memory, and disk usages from 500 containers. Google workload traces [35] contains 28 days of Google usage data workloads consisting of 4,609,3201 tasks comprising CPU intensive workloads, memory-intensive workloads, and both CPU and memory-intensive workloads. The dataset parameters in [35] contain time, job id, parent id, number of cores (CPU workloads), and memory tasks (memory workloads). All experimental data in [34,35] were performed with five-fold cross validation.

For evaluation metrics, we used the Mean Absolute Error (*MAE*), Root Mean Square Error (*RMSE*), Mean Absolute Percentage Error (*MAPE*) and Pearson Correlation Coefficient (*PCC*) to measure the difference between the predicted results and true labels. These three metrics have the property that the smaller the value of *MAE* and *RMSE*, the more the predicted values approach the actual values. The larger the value of *PCC*, the more the predicted values approach the actual values. The definitions of these metrics are given by the following formulas:

(14) $$MAE=\frac{1}{n}\sum_{t=1}^{n}\left|y_{t}-{\bar{y}}_{t}\right|$$

(15) $$RMSE=\sqrt{\frac{1}{n}\sum_{t=1}^{n}{\left(y_{t}-{\hat{y}}_{t}\right)}^{2}}$$

(16) $$MAPE=\frac{100\%}{n}\sum_{t=1}^{n}\left|\frac{{\hat{y}}_{t}-y_{t}}{y_{t}}\right|$$

(17) $$PCC=\frac{\sum \left(y_{t}-{\bar{y}}_{t}\right)\left({\hat{y}}_{t}-{\bar{\hat{y}}}_{t}\right)}{\sqrt{{\sum \left(y_{t}-{\bar{y}}_{t}\right)}^{2}}\sqrt{\sum {\left({\hat{y}}_{t}-{\bar{\hat{y}}}_{t}\right)}^{2}}}$$

where *y_t_* and *ŷ_t_* are the real value and predicted value at time step *t*, respectively; ${\bar{y}}_{t}$ and ${\bar{\hat{y}}}_{t}$ are the real mean value and predicted mean value, respectively; and *n* is the total length of the time steps.

*MSE* was selected as the loss function for the model as:

(18) $$MSE=\frac{1}{n}\sum_{t=1}^{n}{\left(y_{t}-{\hat{y}}_{t}\right)}^{2}$$

The model was trained in Adam optimizer and back-propagation algorithm. The training process is shown in Algorithm 1:
**Algorithm 1** Training process**Input:** *Epoch*, number of trainings iterations. *LR*, learning rate. *Series*, load series. *Label*, ground truth of the prediction.1: *Normseries*←(*Series*-*Series_min_*)/(*Series_max_*-*Series_min_*) 2: *Series Input*←Preprocess(*Normseries*) 3: **For**
*i* in **Epoch do**: 4:          *Prediction*←Model.Forward(*Series Input*) 5:          *MSELOSS*←MSE(*Prediction*, *Label*) 6:        Model.Backward(*MSELOSS*, *LR*) 7: **End** For

After the single and stacked DSTNs were trained, they were verified on the test datasets. The output result passed through the adaptive weight update to produce the predicted result.

### 3.2. Parameter Analyses

#### 3.2.1. Network Layer

**Figure 6:**
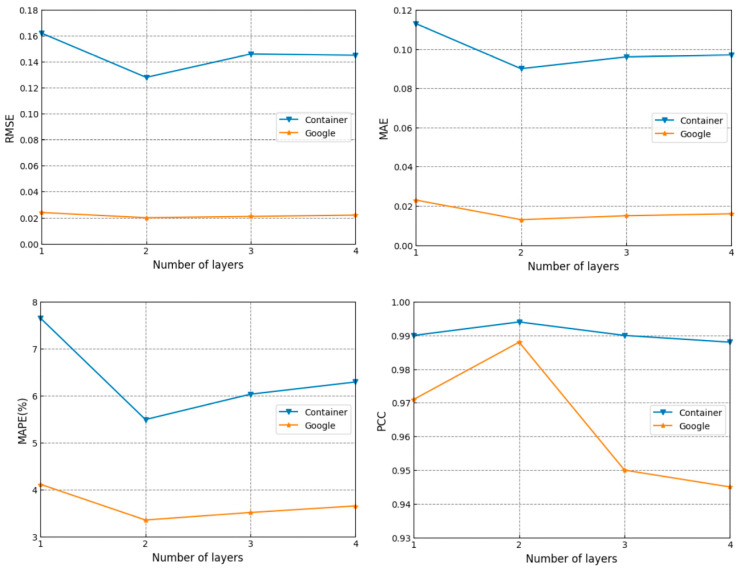
Prediction performance in different layers.

To obtain the best predicted performance, it is necessary to determine the optimal number of layers for the DSTNW. A single and a stacked DSTNs, together as shown in Figure 5, were taken as one network layer. The number of layers changed from one to four with an interval of one. Some experimental results for different layers are given in Figure 6.

One can find from Figure 6 that the performance is the best when the layer is set to two. The performance decreases as the layer increases. Moreover, when the DSTNW contains multiple layers, the network structure becomes complicated and consumes more time. It becomes unable to respond quickly for load predictions. The number of layers was set to two and kept the same for the following experiments.

#### 3.2.2. Time Step

**Figure 7:**
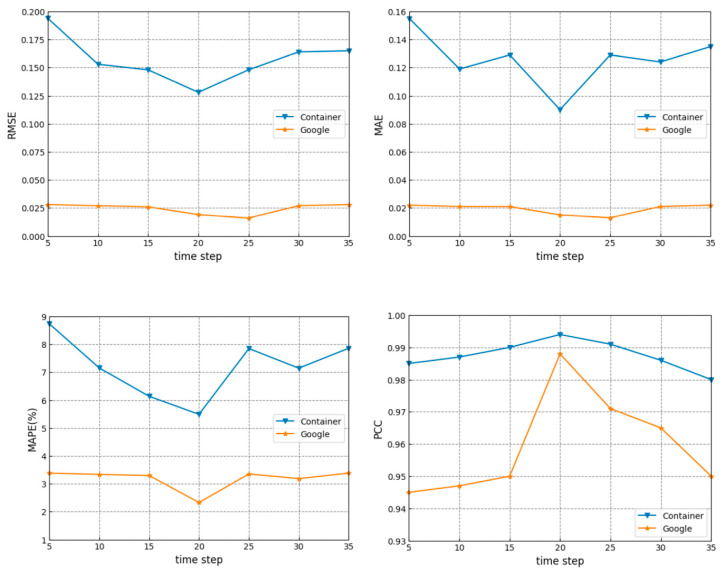
Prediction performance for different time steps *S*.

When the time step *S* is too short, the network is unable to learn effective time information. On the other hand, when *S* is too long, too much redundant information is sent to the network. Too much redundant information hinders the model from learning accurate and efficient advanced representations, which also affects the performance of the network. To obtain a proper time step *S*, we changed the time step *S* from 5 to 35 at an interval of 5. Some experimental results for different time steps *S* are given in Figure 7.

One can find from Figure 7 that the performance is the best when *S* is set to 20. When *S* is less than 20, the prediction performance gradually improves with an increase of *S*. When *S* is larger than 20, the prediction performance begins to degrade as *S* increases. The time step *S* was set to 20 and kept the same in the following experiments.

### 3.3. Ablation Experiment

To evaluate the effectiveness of both DTN and SM, some different models were used to perform experimental tests. Some experimental results are given in Table 1. The optimal results in Table 1 are highlighted in boldface.

One can find from Table 1 that our proposed model and mechanism including the DTN, SM, and DSTNW help to improve the prediction performance, and DSTNW has the best prediction performance. The reason is as follows. The double-channel dilated causal convolution has been adopted to replace the single-channel dilated causal convolution in the developed DTN. Therefore, its prediction performance is superior to that of TCN [26]. Since the SM focuses on features with significant contributions, it helps to improve the network prediction performance. Therefore, the performance of both the TCN-SM and DSTN has been improved to some extent after the TCN [26] and the DTN combined with the SM. Since there are different prediction performances from the single and stacked DSTNs under a complex dynamic cloud environment, an optimal performance is obtained by adaptively assigning different weights to the single and stacked DSTNs. The experimental results showed that the proposed DSTNW has the best performance among the investigated models.

### 3.4. Comparisons with Some State-of-the-Art Methods

Some state-of-the-methods were selected to further evaluate the performance of the DSTNW, including ARIMA [3], LSTM [18], and TCN [26]. To achieve a fair comparison, all corresponding parameters used are the authors’ recommended ones for each method. Some comparative results are given in Table 2. The optimal results in Table 2 are highlighted in boldface.

One can find from Table 2 that the proposed method exhibits the best performance among the selected methods ARIMA [3], LSTM [18], and TCN [26]. Compared with these comparative models, the algorithm we propose has better prediction performance and higher prediction accuracy. The experimental results show that on the Container dataset and the Google dataset, our model has smaller errors in the indicators of *RMSE*, *MAE*, and *MAPE*, and performs better for the *PCC* indicator. The reason is as follows. ARIMA [3] is a linear model in essence, while the cloud load prediction sequence has nonlinear characteristics. LSTM [18] performs poorly in extracting shallow information and is prone to encountering the gradient disappearance problem. This makes it difficult to predict accurately. TCN [26] demonstrates better performance in handling long-term dependencies and has certain advantages in improving generalization and scalability.

Our proposed DSTNW has a stronger generalization ability. It combines single and stacked DSTNs together with an adaptive weight update, which considers more complex dynamic information and extracts deeper network information. A Double-channel Temporal convolution Network model is to capture long-term sequence dependencies and enhance feature extraction capabilities when the model handles long load sequences. A residual temporal self-attention mechanism was proposed to improve the performance of the network and focus on features with significant contributions from the DTN.

In order to explore the training efficiency and convergence performance of each model, the changing trends of the train loss during the training process of the four models on the Container and Google datasets were recorded. The train losses in the figure are the result of training after normalizing the data. On the two datasets, compared with the other three models, the train loss of our model can quickly converge to a smaller value during the training process. It also ends up being stable at a lower point than the others.

**Figure 8:**
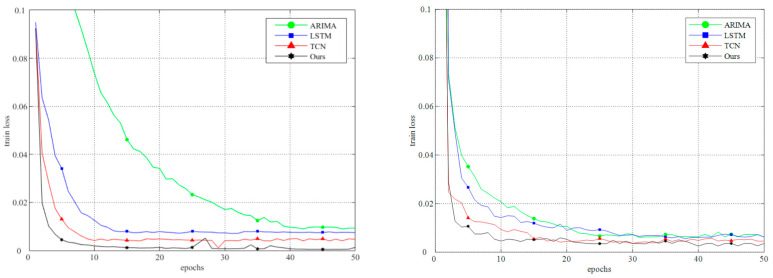
The change trend of the train loss with the number of training epochs on the Container dataset and the Google dataset. The figure on the left is tested on the Container dataset, the figure on the right is tested on the Google dataset.

In order to test the complexity and training time of the proposed approach, a comparative experiment was conducted on the training time of the DSTNW, DSTN, DTN and TCN models. As shown in Figure 8, the experiments used the Container dataset and the Google dataset. The Container dataset and Google dataset used more than 7000 and 5600 datapoints, respectively, for the experiments. The training time was collected and calculated from the beginning of training to the generation of the results. All experimental data were performed with five-fold cross validation. The *t*/*s* values in the figure represent time/second.

Compared with the TCN, the DTN uses dual channels instead of single channels, and the model becomes more complex. Although the model has more calculation steps, the parameter numbers do not increase during the training process of the model. This is why although the computational complexity increases, the training time of the network is only slightly longer. After adding the SM to the DTN, although there is an increase in parameter numbers, the SM module can help the model pay attention to more important features in the sequence, which also makes the training time of the DSTM network not increase, or even decrease. An adaptive weight strategy was adopted in the DSTNW network. This strategy includes the need to train the single and stacked DSTN, as well as weight1 and weight2, which increases the computational complexity, and its training time is also significantly longer.

**Figure 9:**
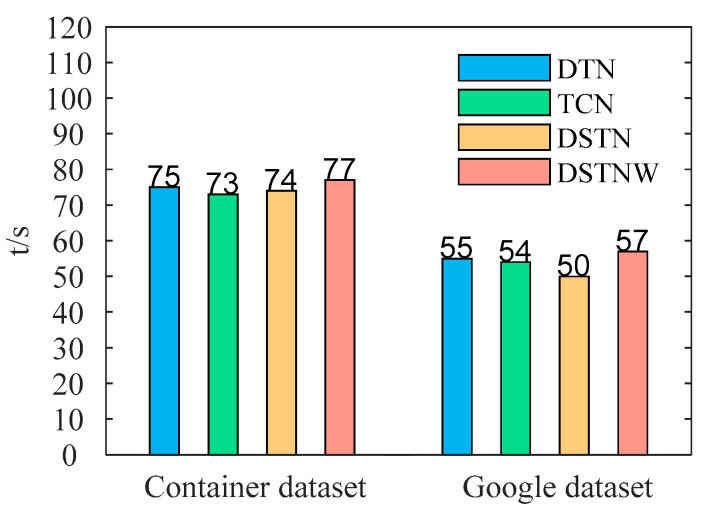
The training time of the DSTNW, DSTN, DTN and TCN models on the Container dataset and the Google dataset.

Although the above Figure 9 shows a significant difference in its training time, this is because we used 5000 pieces of data for comparison in the enlarged experiment. In actual production, its prediction time is much shorter than this, so the difference in their prediction times will also be smaller.

This model is used for resource load prediction in cloud environments. The load prediction result is used for elastically scaling the container in advance. Although the proposed approach has a slight delay compared to the TCN, when we use these methods in a container cluster, the cluster where the DSTNW is located will respond more quickly than the cluster where the TCN is located, and this time difference will be much larger than the model prediction time difference. This is because the prediction accuracy of the DSTNW is higher and the container’s response will be more timely.

## 4. Conclusions

Due to the low feature extraction efficiency of some existing models and complex load environments, load prediction in cloud computing is challenging. A novel cloud computing load prediction model DSTNW has been proposed. It consists of DSTNs with an adaptive weight update strategy. First, double-channel dilated causal convolution was adopted to replace the single-channel dilated causal convolution in DTN. Secondly, the SM was applied to extract the part with the greater contribution in the temporal series. In addition, since it is handling dynamic cloud computing load data in different periods of time, an adaptive weight update strategy was proposed. Some corresponding weights were assigned adaptively to the single and stacked DSTNs. The developed DSTNW has excellent prediction performance for some challenging cloud computing datasets in comparison with the state-of-the-art methods. The proposed method achieved an average improvement of 24.16% and 30.48% on the Container datasets and Google datasets, respectively. This improvement in prediction accuracy has a significant impact on the resource scheduling strategy of cloud computing container clusters, and it can enhance the resource utilization rate of container clusters in the cloud platform.

## Figures and Tables

**Table 1:** Ablation experimental results in different models.

Methods	Container [34]				Google [35]			
	RMSE↓	MAE↓	MAPE↓	PCC↑	RMSE↓	MAE↓	MAPE↓	PCC↑
TCN [26]	0.164	0.124	7.146%	0.988	0.027	0.021	3.186%	0.945
DTN	0.148	0.129	6.138%	0.987	0.026	0.021	3.297%	0.950
TCN-SM	0.153	0.119	7.154%	0.985	0.027	0.021	3.336%	0.947
DSTN	0.148	0.129	7.850%	0.990	0.025	0.015	2.329%	0.971
DSTNW	0.128	0.090	5.491%	0.994	0.020	0.013	2.312%	0.988

Symbol ‘↓’ in Table 1 represents better performance as the value decreases, while symbol ‘↑’ represents better performance as the value increases.

**Table 2:** Experimental comparisons in different methods.

Methods	Container [34]				Google [35]			
	RMSE↓	MAE↓	MAPE↓	PCC↑	RMSE↓	MAE↓	MAPE↓	PCC↑
ARIMA [3]	0.194	0.155	10.235%	0.975	0.029	0.023	3.502%	0.948
LSTM [18]	0.168	0.129	8.413%	0.976	0.028	0.022	3.383%	0.944
TCN [26]	0.164	0.124	7.146%	0.988	0.027	0.021	3.186%	0.945
Ours	0.128	0.090	5.491%	0.994	0.020	0.013	2.312%	0.988

Symbol ‘↓’ in Table 2 represents better performance as the value decreases, while symbol ‘↑’ represents better performance as the value increases.

## Data Availability

All datasets are available in the Internet Traffic Archive https://github.com/Hardy-linjiang/datasets (accessed on 5 January 2024).
